# Illumina sequencing of complete chloroplast genome of *Avicennia marina*, a pioneer mangrove species

**DOI:** 10.1080/23802359.2020.1768927

**Published:** 2020-05-20

**Authors:** Huan Li, Dongna Ma, Jing Li, Mingyue Wei, Hailei Zheng, Xueyi Zhu

**Affiliations:** Key Laboratory of the Ministry of Education for Coastal and Wetland Ecosystems, College of the Environment and Ecology, Xiamen University, Xiamen, China

**Keywords:** *Avicennia marina*, mangrove, chloroplast genome, illumina sequencing, phylogenetic analysis

## Abstract

Mangrove tree *Avicenna marina* has great ecological significance in maintaining coastal ecosystem, but its unique potential for gene functions and genetic diversity underlying ecological adaptation remains investigation. In this study, the chloroplast genome of *A. marina* was first characterized by sequencing chloroplast DNA with Illumina technology. The *A. marina* genome was 147,909 bp in size with a typical quadripartite structure, which was deposited in GenBank under the accession number MT108381.

## Introduction

*Avicennia marina*, a pioneer mangrove species with the strong wind control and the higher ability of salt tolerance in China (Liao and Zhang 2014). It plays a significant role in supporting coastal ecosystem and holds unique potential for studying molecular mechanisms underlying ecological adaptation.

The chloroplast genomes contain highly conserved essential genes for plant growth and development (Wicke et al. 2011), but they also harbor a variety of genetic polymorphisms (Daniell et al. 2016; Sakaguchi et al. 2017). The whole chloroplast genome data obtained from next-generation sequencing technology offer promise for further researches in chloroplast gene functions and genome diversity.

The fresh leaves of *A. marina* were collected in August 2018 from Zhangjiang Estuary, a Mangrove National Nature Reserve (23°55′N, 117°26′E), Fujian, China. Voucher specimen was kept in the Herbarium of School of Life Sciences, Xiamen University with specimen code XYZ20180818. The intact chloroplasts were isolated with a high salt isolation buffer followed by a saline Percoll gradient centrifugation, from which the chloroplast DNA (cpDNA) was isolated according to Vieira et al. (2014). Illumina sequencing produced 7,672,820 paired-end raw reads with an Illumina HiSeq TM2000 platform. Filtered 6,445,660 chloroplast genome reads (949,811,469 bp) were used to constructure the chloroplast genome by the NOVOPlasty program (version 3.3) with *Aloysia citrodora* (GenBank: NC_034695) as a reference (Dierckxsens et al. 2017). The web-based program GeSeq Annotator (https://chlorobox.mpimp-golm.mpg.de/geseq.html) was used to annotate protein coding genes and ribosomal RNA genes. The transfer RNA genes were identified with GeSeq and the tRNAscan-SE program v2.0.3 (Schattner et al. 2005). Circular representation of the chloroplast genome was created by the web-based program OGDRAW tool (http://ogdraw.mpimp-golm.mpg.de/) (Lohse et al. 2013).

The complete chloroplast genome of A. marina with a typical quadripartite structure was 147,909 bp in size (38.15% CG content), which consisted of a small single copy region (SSC) of 17,772 bp, a large single copy region (LSC) of 88,331 bp, and a pair of inverted repeats (IRs) of 20,903 bp in size. There were 112 genes in total, including 78 protein-coding genes, 30 tRNA genes, and 4 rRNA genes, of which 14 genes occurred in IRs, containing 5 protein-coding genes, 5 tRNA genesand 4 rRNA genes. In addition, there were 10 intron-containing genes, nine protein-coding genes and one tRNA genes.

**Figure 1.**
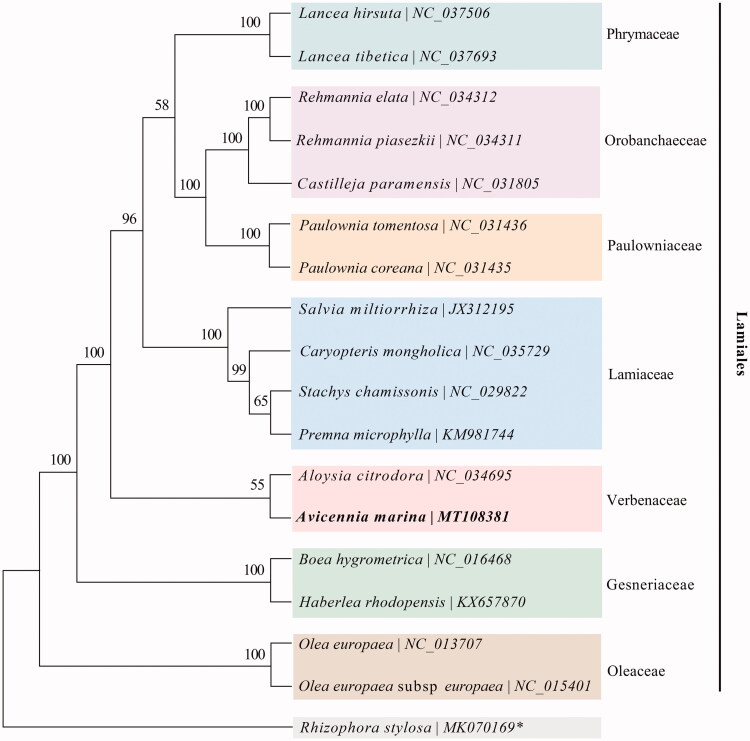
Phylogenetic tree conducted with Maximum Likelihood (ML) method based on 76 protein-coding genes shared between 17 chloroplast genomes under the order Lamiales and 1 outgroup marked by asterisks (*).

To confirm the phylogenetic position of this newly described chloroplast genome, the 16 chloroplast genomes under the order Lamiales and one mangrove species *Rhizophora stylosa* (as an outgroup) from NCBI were used to build the phylogenetic tree based on 76 protein-coding genes, which were aligned with MAFFT and concatenated with Gblocks (Nakamura et al., 2018). Phylogenetic reconstruction was based on Maximum Likelihood analysis with GTR + G nucleotide substitution Model and 1000 bootstrap replicates using the MEGA X (Kumar et al. 2018). The phylogenetic analysis confirmed that *A. marina* was grouped into the family of Verbenaceae (Figure 1).

## Data Availability

The data that support the findings of this study are openly available in NCBI GenBank database at https://www.ncbi.nlm.nih.gov/nuccore/MT108381.
